# Adjustment errors of sunstones in the first step of sky-polarimetric Viking navigation: studies with dichroic cordierite/ tourmaline and birefringent calcite crystals

**DOI:** 10.1098/rsos.150406

**Published:** 2016-01-20

**Authors:** Dénes Száz, Alexandra Farkas, Miklós Blahó, András Barta, Ádám Egri, Balázs Kretzer, Tibor Hegedüs, Zoltán Jäger, Gábor Horváth

**Affiliations:** 1 Environmental Optics Laboratory, Department of Biological Physics, Physical Institute, Eötvös University, Pázmány sétány 1, Budapest 1117, Hungary; 2 Danube Research Institute, MTA Centre for Ecological Research, Karolina út 29–31, Budapest 1113, Hungary; 3 Estrato Research and Development Ltd, Nemetvolgyi ut 91/c, Budapest 1124, Hungary; 4 Astronomical Observatory of Baja, University of Szeged, Pf. 766, Baja 6500, Hungary

**Keywords:** Viking navigation, sunstone, dichroism, birefringence, compass direction, sky polarization

## Abstract

According to an old but still unproven theory, Viking navigators analysed the skylight polarization with dichroic cordierite or tourmaline, or birefringent calcite sunstones in cloudy/foggy weather. Combining these sunstones with their sun-dial, they could determine the position of the occluded sun, from which the geographical northern direction could be guessed. In psychophysical laboratory experiments, we studied the accuracy of the first step of this sky-polarimetric Viking navigation. We measured the adjustment error *e* of rotatable cordierite, tourmaline and calcite crystals when the task was to determine the direction of polarization of white light as a function of the degree of linear polarization *p*. From the obtained error functions *e*(*p*), the thresholds p* above which the first step can still function (i.e. when the intensity change seen through the rotating analyser can be sensed) were derived. Cordierite is about twice as reliable as tourmaline. Calcite sunstones have smaller adjustment errors if the navigator looks for that orientation of the crystal where the intensity difference between the two spots seen in the crystal is maximal, rather than minimal. For higher *p* (greater than *p*_crit_) of incident light, the adjustment errors of calcite are larger than those of the dichroic cordierite (*p*_crit_=20%) and tourmaline (*p*_crit_=45%), while for lower *p* (less than *p*_crit_) calcite usually has lower adjustment errors than dichroic sunstones. We showed that real calcite crystals are not as ideal sunstones as it was believed earlier, because they usually contain scratches, impurities and crystal defects which increase considerably their adjustment errors. Thus, cordierite and tourmaline can also be at least as good sunstones as calcite. Using the psychophysical *e*(*p*) functions and the patterns of the degree of skylight polarization measured by full-sky imaging polarimetry, we computed how accurately the northern direction can be determined with the use of the Viking sun-dial under 10 different sky conditions at 61° latitude, which was one of the main Viking sailing routes. According to our expermiments, under clear skies, using calcite or cordierite or tourmaline sunstones, Viking sailors could navigate with net orientation errors $|\Sigma_{\max}|\leq 3^{\circ }$. Under overcast conditions, their net navigation error depends on the sunstone type: $|\Sigma_{\max}(\text{calcite})|\leq 6^{\circ }$, $|\Sigma_{\max}(\text{cordierite})|\leq 10^{\circ }$ and $|\Sigma_{\max}(\text{tourmaline})|\leq 17^{\circ }$.

## 1. Introduction

In the ninth to eleventh centuries, the Vikings were prominent sailors and experienced navigators [1–3]. During their coastal journeys, they might have used natural navigational signals (e.g. hills and mountains, bays and islands, trees and cairns) and the habitat borders of marine animals (e.g. whales, birds) to orient themselves [4]. It is still a mystery, how they could navigate on the open sea without reliable reference directions and a magnetic compass. They could take advantage of atmospheric optical navigation cues, such as crepuscular rays [5,6] or arctic mirages [7], for example.

The only clue to solve the mystery of Viking navigation is a fragment of a wooden dial found in Greenland in 1948, under the ruins of a Benedictine convent in an ancient Viking colony, near the fjord of Uunartoq. This fragment turned out to be a legacy from the ninth century. In later decades, this artefact was in the crossfire of debates about its function and usage. According to the most accepted explanation, this fragment was part of a special sun-compass. This theory is confirmed by the deliberately carved lines on the compass surface. Other arguments for and against the possible function(s) of the fragment have also been described [8–12]. This instrument was possibly usable even right after sunset and before sunrise, when the sun was under the horizon [13]. Alternatively, it was interpreted as an instrument to determine latitude and local noon [14].

Using a sun-compass, the Vikings could easily find the geographical northern direction with high precision, but only in clear weather. In the Viking sailing routes, however, the sun was often covered by clouds or fog. According to a widely accepted theory, in such situations, the Vikings had special crystals, called sunstones, through which they could detect the direction of polarization of skylight, which proved to be a good basis of navigation. According to the famous Sigurd's story of the saga of King Olaf, the Holy, such a sunstone allowed him to see the occluded sun [15]. There was, however, no detailed description of its use, only some mentioning in treasure inventories proved the high value of the sunstone crystals [16]. According to the most widespread hypothesis, the sunstones were used for skylight polarimetry: analysing the celestial pattern of the direction of polarization, Viking navigators could locate the position of the sun even under cloudy or foggy conditions [17–19]. With the combination of sunstones and the sun-compass, one gets a device which could have enabled the Vikings to determine the North direction under any weather conditions throughout the day. When the sun was occluded, sunstones could have been used to determine the sun position, and a shadow-stick might have been used as a shadow-replacement (e.g. [13,20]). Thus, the Vikings could navigate until the sky was bright enough to see its polarization pattern through the sunstones. Furthermore, the navigation could become possible even after sunset and before sunrise with a twilight board toolkit [13].

The identity of Viking sunstones is strongly debated. Ramskou [18,21] suggested that the Viking sunstones described in the old sagas could have been dichroic cordierite, andalusite and tourmaline or birefringent calcite (Iceland spar) crystals that could serve as linear polarization analysers. These crystals can be found on the beaches of Norway and Iceland where the Vikings lived. These hypotheses have been frequently cited [19,22–33]. A calcite crystal was found in a shipwreck at Alderney, which raised the possibility that such crystals were used for navigational purposes even in the sixteenth century [34]. If one side of the calcite is covered so that only one narrow slot or spot remains uncovered, the optical image doubles on the opposite side due to birefringence (see figs. 2–4 in [20,35,36]). The directions of polarization of the two neighbouring slots/spots are perpendicular to each other. The hypothesized steps of sky-polarimetric Viking navigation are the following (see fig. 1 in [37]):

— *Calibration step*: In cloudless weather, the navigator (being always a male) watched the sky through a sunstone, and while rotating it to and fro in front of his eyes, he could detect periodic changes in the intensity of transmitted skylight. He had to rotate (adjust) the crystal until its well-determined orientation (e.g. minimal or maximal intensity of skylight transmitted through a dichroic sunstone, or minimal or maximal intensity difference between the two slots/spots of a birefringent sunstone), where it was fixed, and thereafter he calibrated the crystal by engraving the direction pointing towards the sun on the crystal surface.
— *Navigation step 1*: Applying this sunstone rotational adjustment under a cloudy or foggy sky at two different celestial points, the navigator could determine the directions perpendicular to the local E-vectors of skylight shown by the engraved straight markings of the sunstones, pointing towards the sun.
— *Navigation step 2*: The intersection of the two celestial great circles crossing the sunstones parallel to their engravings gives the position of the invisible sun.
— *Navigation step 3*: Using the Viking sun-compass, the navigator could derive a true compass (e.g. North) direction from the estimated position of the invisible sun [18,37].

**Figure 1.**
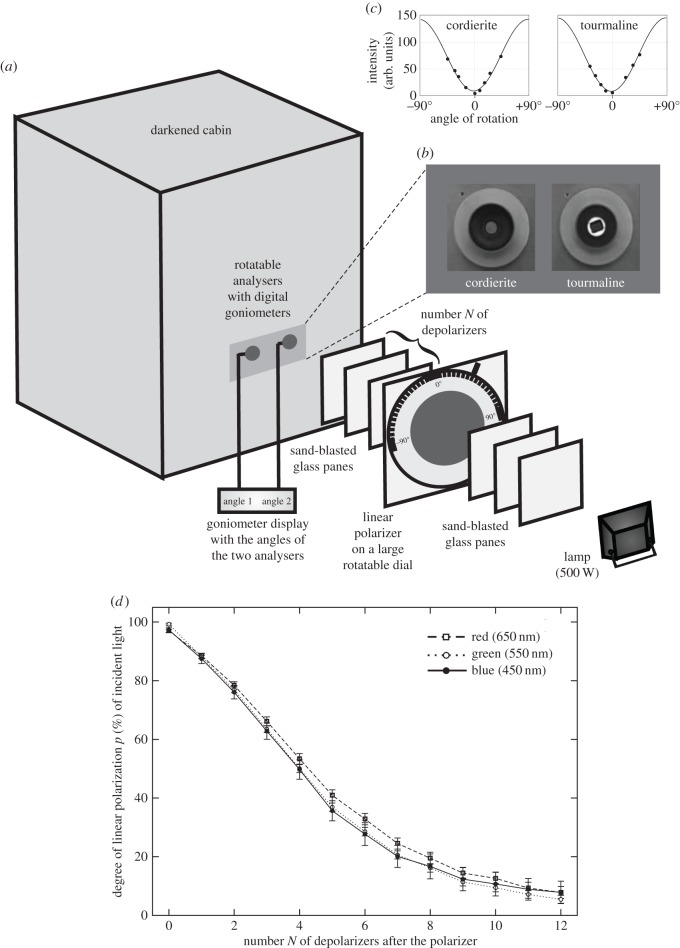
(*a*) Schematics of the experimental set-up (more details in the text). The number *N* of depolarizers between the polarizing dial and the analysers (cordierite, tourmaline) ranged between 0 and 12. (*b*) The rotatable cordierite and tourmaline minerals as seen by the test persons from the darkened cabin. (*c*) Change of the intensity of light transmitted through the analysers as a function of the angle of rotation. Dots: measured intensity values of green light transmitted by a fixed linear polarizer. Continuous curve: sinusoid function fitted to the measured data (dots) with the method of least squares. All samples of both crystals are equally effective polarizers. (*d*) Average (dots) and standard deviation (vertical T-shaped bars) of the degree of linear polarization *p* of light illuminating the analysers (cordierite and tourmaline) in our experiment as a function of the number *N* of sand-blasted glass depolarizers between the polarizing dial and the analysers (figure 1*a*) measured by imaging polarimetry in the red (650 nm), green (550 nm) and blue (450 nm) parts of the spectrum.

Inevitably, every step has certain errors, the cumulation of which can spoil the accuracy of this navigation method causing the navigators to get lost or to deviate from the original seafaring route. In addition, the degree *p* of skylight polarization depends strongly on the meteorological circumstances [38–41], and highly decreased *p* values enhance the errors of the determination of the sun position. The atmospheric optical prerequisites of this sky-polarimetric Viking navigation have been studied [42,43].

Earlier field experiments demonstrated that birefringent calcite sunstones are undoubtedly effective in the analysis of sky polarization [13,14]. A planetarium experiment investigated the accuracy of the second step of sky-polarimetric Viking navigation, i.e. how accurately the navigator could determine the intersection of two celestial great circles [37]. We present here the results of our psychophysical laboratory experiment in which we measured the earlier unknown errors of the first step of sky-polarimetric Viking navigation, i.e. how accurately the navigator could adjust sunstone crystals such as dichroic cordierite and tourmaline or birefringent calcite to the right orientation to determine the direction of skylight polarization. In a pilot experiment, we experienced that the errors of sky-polarimetric Viking navigation strongly depend on the quality (especially on impurities and crystal defects) of the calcite crystal used. Thus, we performed our experiment with four calcite crystals of different qualities. Using the measured adjustment errors, we calculated the errors of North determination (i.e. the deviations from geographical North), assuming that the further (second and third) steps of sky-polarimetric Viking navigation were errorless. The results presented here are essential to answer the question: How accurately could the Vikings navigate with cordierite, tourmaline or calcite sunstones under different meteorological circumstances on the open sea?

## 2. Material and methods

### 2.1 Experimental set-up and procedure

#### 2.1.1 Experiment 1

Experiment 1 was conducted with 10 male test persons (aged between 24 and 63 years) in the Environmental Optics Laboratory of Eötvös University (Budapest, Hungary). The experimental set-up (figure 1*a*) consisted of a 500-W incandescent lamp producing intense white light (corresponding to sunlight), a linear polarizer (diameter=40 cm, thickness=1 mm, type: XP42–18 from ITOS, Mainz, Germany) on a large (diameter = 50 cm) rotatable dial (representing the variable direction of polarization of skylight), 12 sand-blasted, colourless glass panes (500×500×4 mm) as diffusing depolarizers (with which we simulated the different degrees *p* of skylight polarization of the same intensity under various weather conditions), and two rotatable metal sockets (figure 1*b*,*c*, diameter=5 cm) with a cordierite and a tourmaline crystal (modelling dichroic sunstones) placed on a metal panel on the wall of a darkened cabin (150×150×200 cm) covered inside with a depolarizing matt white cloth. The optical device and the cabin were in a darkened laboratory room with matt white walls. The test person sat in the cabin, where no outer light sources disturbed him, and the weak light reflected from the matt white cabin wall was unpolarized. The adjusted orientations of the dichroic sunstone crystals were registered with digital goniometers, the accuracy of which was ±0.5°.

The angle (direction) of polarization of the practically totally linearly polarized light with *p*=99.98% produced by the polarizing dial could be adjusted with an accuracy of ±0.5° between 0° and 180° with respect to the horizontal. This totally polarized light was more or less depolarized after it passed through some diffusing screens (depolarizers: wooden-framed glass panes, both sides of which were sand-blasted and one glass pane with only one sand-blasted side). In the light path, there were always 12 depolarizers. When all 12 depolarizers were placed between the lamp and the polarizing dial, the analysers were illuminated by totally polarized light (*p*≈100%). Placing more and more (*N*=0,1,2,…,12) depolarizers from the lamp-side of the polarizing dial to its other side (figure 1*a*), the *p* of light illuminating the analysers decreased gradually, stepwise (if *N*=0, 1, 2, 3, 4, 5, 7, 9, 11, 12, then *p*=99.26, 88.08, 76.88, 63.48, 50.02, 36.88, 20.61, 11.36, 7.14, 5.45 %). When all 12 depolarizers were between the polarizing dial and the analysers, the latter were illuminated by practically unpolarized light (*p*=5.5%). Since always 12 depolarizers were in the light path, the intensity and spectrum of light incident onto the analysers was constant, independently of *p*. With this depolarization method, we could simulate the reduced degrees of skylight polarization occurring under different meteorological conditions: the average degrees of polarization of cloudy and foggy skies, for example, are *p*_cloudy_=10–25% and *p*_foggy_=4–15% [41]. Using sand-blasted glass panes as depolarizers has the advantage that glass is optically inactive, so that is it does not change the state of polarization of transmitted light. Using imaging polarimetry [39,44], we measured the degree *p* and angle *α* of polarization of light incident on the rotatable analysers in the red (650 nm), green (550 nm) and blue (450 nm) spectral ranges as a function of the number *N* of depolarizers between the polarizing dial and the analysers (figure 1*d*). The average of *α* was practically constant, but its standard deviation slightly increased with decreasing *p*.

The rectangular cordierite and tourmaline fragments (12×8×1 mm) were split from larger crystals (in the Department of Mineralogy of Eötvös University, Budapest) so that they functioned as ideal linear polarizers (figure 1*c*). Obviously, Viking navigators could not use such ideal crystals, but could apply similar crystals of poorer quality.

#### 2.1.2 Experiment 2

Experiment 2 was performed with four different calcite crystals as sunstones, and 11 male test persons (aged between 22 and 65 years) were involved. Apart from two, these persons were the same as in experiment 1. The experimental set-up and procedure were the same as used in experiment 1. The calcite crystals had different opacities (calcites 1 and 4 had more surface scratches than calcites 2 and 3) and colour (calcites 1 and 4 were white, while calcites 2 and 3 were yellowish). Their thickness was 22 mm with the same length of optical path of transmitted light. Calcites 2 and 3 were split from the same major crystal with the difference that the surface of the latter was polished. One side of the calcite crystals was covered with a black cardboard, from which a small hole with a diameter of 3 mm was cut out. After placing the crystals in the analyser sockets, a double-image of the holes was formed due to birefringence of calcite. The direction of polarization of these two light spots was perpendicular to each other. The calcite crystals were intentionally different, because we wanted to simulate that the Vikings might have used sunstones of different optical properties.

### 2.2 Tasks of test persons in the experiments

In experiment 1, 10 test persons were involved, each doing the same measurement series 10 times. Thus, we performed altogether 100 series of measurements. In every measurement session, the task of the test person was to adjust sequentially the analysers into a specific orientation: the cordierite crystal and then the tourmaline crystal had to be rotated until the lowest intensity of light transmitted through them was found. The search of minimal (rather than maximal) transmitted intensity was motivated by the fact that Viking navigators used the sunstone under a bright sky: in front of a bright background (sky), it is easier to find the darkest state of a light spot (rotating dichroic sunstone) than the brightest one. After each session, the experiment leader changed both the degree *p* and angle *α* of polarization of the incident white light of constant intensity. One measurement session consisted of 10 different (*p*, *α*) pairs modelling 10 different states of skylight polarization.

One of our goals was to determine the lowest p* value at which the test persons can still detect the maxima and minima of the sinusoidally changing intensity of light transmitted through the rotating dichroic sunstone crystals. Thus, we asked the test persons to signal whether they could or could not see intensity changes through the rotating analysers (table 1).

**Table 1.** Fraction *f* of the total number of cases (*n*=100) when the test persons could not sense intensity changes seen through the rotating dichroic cordierite and tourmaline as functions of the number *N* of depolarizers (between the polarizing dial and the analysers) and the degree of linear polarization *p* (%) of transmitted light in the green (550 nm) part of the spectrum in which the human eye is the most sensitive. Cases with *N*=6, 8 and 10 do not occur, because in our experiment we used only 0, 1, 2, 3, 4, 5, 7, 9, 11 and 12 depolarizers between the polarizing dial and the analysers.

number *N* of depolarizers	0–5	7	9	11	12
*p*_green_ (%)	99.3–36.9	20.6	11.4	7.1	5.5
*f* (cordierite)	0	0.03	0.10	0.31	0.39
*f* (tourmaline)	0	0.02	0.20	0.58	0.64

**Figure 2.**
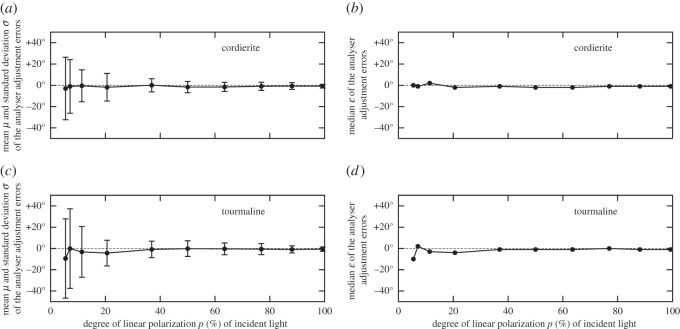
Mean *μ* with standard deviation *Σ* (*a*,*c*) and median *ε* (*b*,*d*) of the adjustment error angles measured for the cordierite (*a*,*b*) and tourmaline (*c*,*d*) crystals in our experiment 1 as a function of the degree of linear polarization *p* (%) of incident light averaged for the 100 measurements performed with 10 test persons.

The experiment leader read the orientations (angles from the horizontal) of the adjusted analysers and the polarizing dial on digital goniometers. The obtained data were evaluated with a self-written computer program providing the following parameters (figure 2): (i) mean *μ* with standard deviation *Σ* and median *ε* of the adjustment angle errors of the analysers averaged for the 10 measurements for each test person, and (ii) *μ*, *Σ* and *ε* averaged over the 100 measurements performed with the 10 test persons.

**Figure 3.**
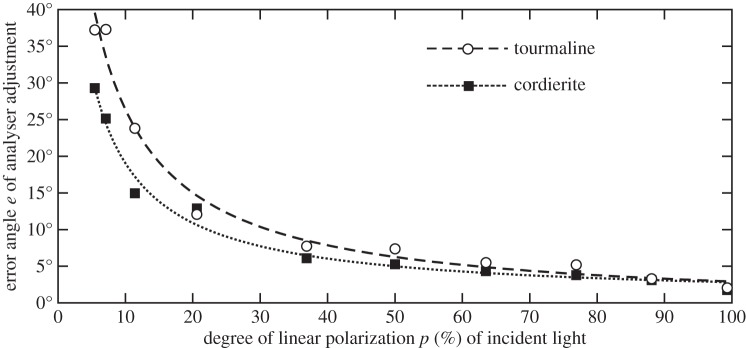
Error functions *e*(*p*) obtained for the dichroic cordierite and tourmaline crystals in our experiment 1 averaged for the 100 measurements performed with 10 test persons. *p* (%) is the degree of linear polarization of incident light, and *e* is equal to the standard deviation *Σ* of the error angles of analyser adjustment. The continuous *e*(*p*) curves are hyperbolas fitted to the measured data points.

After getting the mean errors *μ* for the analysers, we plotted their standard deviations *Σ* versus *p* giving the maximal possible navigation error by which a navigator could be mistaken under a specific weather condition with a given degree of skylight polarization *p*. Since we had errors *e*_*i*_=*Σ*_*i*_ only for some discrete *p*_*i*_ values (*i*=1,2,…,10), we fitted a hyperbolic function *e*(*p*) to these data points (*e*_*i*_=*Σ*_*i*_, *p*_*i*_) obtaining the error function *e*(*p*) for each analyser (figure 3). As the degree of polarization *p* can never be negative (*p*≥0), we used only the hyperbola part above the horizontal asymptote. The other part (below the asymptote) can be ignored, since *p*<0 does not exist in our case. Although the error function *e*(*p*) is bounded (where the maximal possible error is 90° for both the cordierite and tourmaline), the *p* values at which the fitted functions reach these boundary values are *p*=0.04% for cordietite and 0.39% for tourmaline. These values are far below the threshold above which the test persons could perceive intensity changes in the sunstone crystals. Furthermore, for the North error determination, we excluded sky regions with *p*<5% (being under the sensitivity threshold of the human eye to distinguish intensity changes produced by rotating analysers according to the results of our present experiment, table 1, figures 2 and 3) from computation (this exclusion was achieved during data processing, rather than physically in the experiment). Degree of polarization values *p*>90% can be induced by motional artefacts [39] caused by moving clouds or objects if the polarization pictures necessary for imaging polarimetry are taken sequentially, rather than simultaneously. Since *p*>90% values normally do not occur in real skies, we excluded also these very high *p* values during data processing. Consequently, in all the studied cases, the fitted hyperbolic functions represented well the error function *e*(*p*) for 5%<*p*<90%.

In every measurement of experiment 2, the test person had to adjust one of the four calcite crystals to a specific orientation. (i) Task 1 (equal intensity): the crystal had to be rotated until the same intensity of the two light spots was seen. (ii) Task 2 (maximal contrast): the crystal had to be rotated until the maximal difference between the intensities of the two spots was found. We expected that the angle of polarization of incident light can be determined with different accuracies using these two adjustment tasks. After the test person adjusted one calcite analyser, the experiment leader changed randomly the angle *α* of polarization of the incident white light of constant intensity and spectrum. After measuring all four calcite crystals, the experiment leader changed randomly the degree *p* of polarization of incident light. One measurement session consisted of the measurement of all four calcites with 10 different *p* values of the incident light. During one task (1 or 2), the same measurement session was repeated 10 times. Thus, altogether 220 measurement sessions were performed with the 11 test persons for every *p* value of incident light.

We asked the test persons to signal weather they could see any intensity change of the two light spots while rotating the calcite crystals. Thus, we could determine the threshold p* at which the test persons were still able to consciously perceive intensity changes while rotating the sunstones. The thresholds p* obtained for the two tasks were compared to get information on which adjustment method (equal intensity or maximal contrast) results in a higher accuracy. In tasks 1 and 2, threshold p* was determined for 10% and 50% of the 110 meaurements at a given *p* value, above which threshold (*p*>p*) we consider that intensity changes cannot be seen between the two light spots.

### 2.3 Determination of the error function in experiment 2

The evaluation of the measurement data of experiment 2 was performed in the same way as in experiment 1. However, because of surface scratches and impurities/defects in the calcite crystals, we had to solve the following problem: due to birefringence, in calcite there are two optical paths. If the scratches/impurities/defects (called contamination further on) in the two paths are different, the absorption, scattering and internal reflections of light along the two paths can also be different. This influences the accuracy of adjustment of the calcite crystals in tasks 1 and 2.

In task 1, the calcite is rotated until the intensities of the two light spots are equal. During a full 360° rotation, there are also four orientations where the two intensities are equal. If the calcite contaminations between the two optical paths are equal, these four angles of equal intensities are independent of the degree of polarization *p* of incident light and are 90° apart from each other. However, if there is a difference in the contamination between the two optical paths, the equal intensity angles depend on *p*. Furthermore, there is a threshold *p*^+^ below which the light spot belonging to the less contaminated optical path is always brighter than the other (electronic supplementary material, figure S1). In this case, the navigator cannot scratch a single sun mark onto the sunstone, since the direction of the sunmark would depend on *p* making this method practically unusable for a Viking navigator.

To temporarily overcome this issue in order to still be able to determine the accuracy of finding the equal intensity angles of the crystals, we previously measured the intensity of light seen through the spots for each calcite crystal: for every degree of polarization *p*, in every 10° rotation of the calcite we measured the intensities of the two light spots. Then, we determined the intensity difference of the two light spots: Δ*I*=*I*_spot1_−*I*_spot2_. Where this difference was zero, that angle was considered as the exact equal intensity angle. To the measured intensity difference data, we fitted the function

1 y(x)=a⋅sin⁡(2x+b)+c,

where *y* is the intensity difference, *x* is the angle rotated from the reference direction, *a*, *b* and *c* are fitting parameters. Using *y*(*x*), we determined the equal intensity angles, from which we determined the adjustment errors of the test persons.

In task 2, the calcite is rotated until the intensity difference between the two light spots is maximal. This occurs four times with 90° periodicity during a full 360° rotation of the crystal. Angles where the intensity difference is maximal are independent of the degree of polarization of incident light and the disturbing differences in calcite contamination between the two optical paths. Therefore, if during sunstone calibration the Viking navigator uses this maximal contrast method, he has to scratch only one straight mark (pointing towards the sun) onto the sunstone, and this sun mark can be used under all weather conditions to determine the position of the invisible sun.

**Figure 4.**
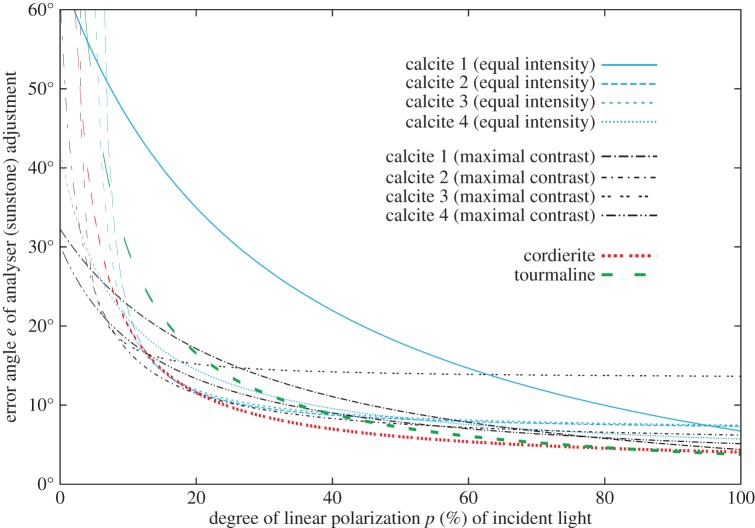
Error functions *e*(*p*) obtained for the four birefringent calcite crystals in our experiment 2 averaged for the 110 measurements of task 1 (equal intensity adjustment) and task 2 (maximal contrast adjustment) performed with the 11 male test persons. As comparison, the error functions of dichroic cordierite and tourmaline crystals (figure 3) are also displayed. *p* (%) is the degree of linear polarization of incident light, and *e* is the sum of the mean *μ* and standard deviation *Σ* of the error angles of sunstone (analyser) adjustment. The continuous hyperbolic *e*(*p*) curves were fitted to the measured data points.

After getting the adjustment errors, we characterized the accuracy of calcite adjustment with the mean *μ* and standard deviation *Σ* of errors of the 110 measurements for every *p* value of the incident light and for both tasks. For each calcite crystal, we determined the error function *e*(*p*) by fitting a hyperbolic function to the values of *μ*+*Σ* (figure 4). We used this sum because the standard deviation *Σ* can only be considered as the error if the mean error *μ* is zero. However, if *μ* differs from zero, it gives an additional error in the sunstone adjustment.

When in task 1 (equal intensity) the degree of polarization *p* of incident light was so low (*p*<*p*^+^) that no equal intensity angle occurred, we assumed that the test person rotated an ideal calcite (without contaminations) randomly with no systematic error, which could result in an error between 0° and 45°. Considering a uniform distribution and no systematic error, the expected error value is 22.5°. Thus, *e*(*p*<*p*^+^)=22.5°, while for *p*>*p*^+^ the error function *e*(*p*) was calculated according to the hyperbolic fit. With a systematic error, the mean could exceed 22.5°.

### 2.4 Determination of the North error of sky-polarimetric Viking navigation

**Figure 5.**
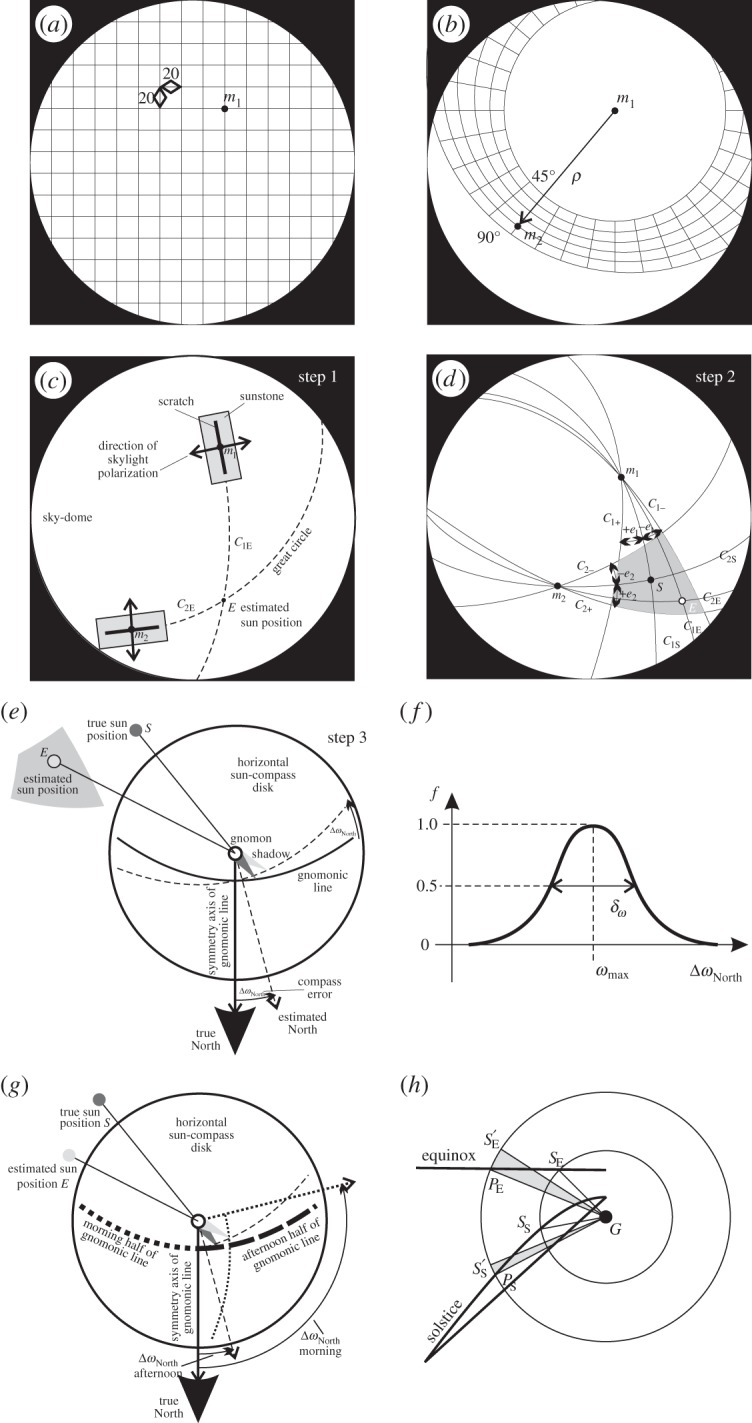
Steps of determination of the North error Δ*ω*_North_. (*a*) Celestial square grid from which sky point *m*_1_ is chosen, where the first sunstone is rotated. (*b*) Polar grid from which point *m*_2_ is chosen, where the second sunstone is rotated at an angular distance *δ* from *m*_1_. (*c*) The first step of sky-polarimetric Viking navigation. (*d*) Adjusting the orientation of sunstones at sky points *m*_1_ and *m*_2_ has errors *e*_1_ and *e*_2_, which determine a spherical tetragon (grey) containing the real sun position *S* and all possible estimated sun positions *E*. (*e*) The third step of sky-polarimetric Viking navigation with a North error Δ*ω*_North_. (*f*) Distribution (frequency *f*) of the North error Δ*ω*_North_ with a maximum at angle $\omega_{\max}$ and half bandwidth *δ*_*ω*_ being the full width at half maximum. (*g*) The two possibilities to project the estimated sun position *E* onto the morning and afternoon half of the gnomonic line. (*h*) The gnomonic lines for the spring equinox (21 March) and the summer solstice (21 June), onto which the real and estimated sun positions were projected. The angular deviation from the gnomonic lines gives the navigational error. G: gnomon. *S*_E_: real sun position projected onto the equinoctial line. *S*′_E_: estimated erroneous sun position projected onto the equinoctial line. *P*_E_: point that we get after rotating *S*′_E_ to fit onto the equinoctial line (the main step of North error computation). *S*_S_: real sun position projected onto the solstice line. *S*′_S_: estimated erroneous sun position projected onto the solstice line. *P*_S_: point that we get after rotating *S*′_S_ to fit onto the solstice line (the main step of North error computation). Grey: angles Δ*ω*_North_ with which *S*′_E_ and *S*′_S_ need to be rotated to fit onto the equinoctial and solstice line, respectively. More details can be read in the text.

**Figure 6.**
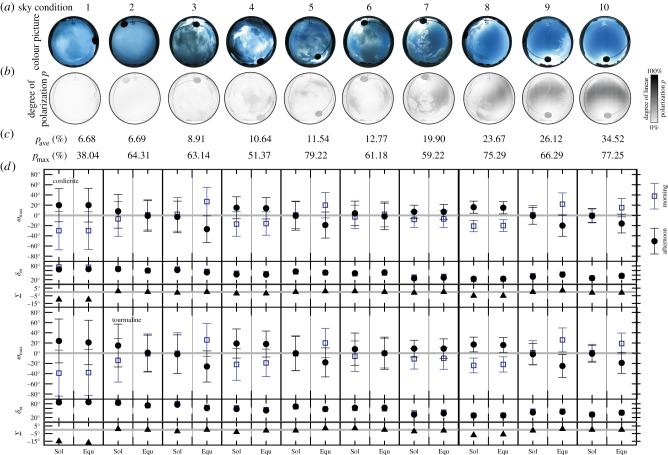
Accuracy of the North determination derived from the measured error function *e*(*p*) of the dichroic cordierite and tourmaline crystals calculated for 10 different sky conditions. (*a*) 180° field-of-view colour picture of the sky photographed under different weather conditions without a polarizer. (*b*) Patterns of the degree of linear polarization *p* of skylight (white: *p*=0%; black: *p*=100%; the darker the grey, the higher the *p*) measured by imaging polarimetry in the green (550 nm) spectral range. Only celestial areas with 5%<*p*<90% were taken into account during the computation of the North error. (*c*) The average *p*_ave_ and maximum $p_{\max}$ of *p* calculated for sky regions with 5%<*p*<90%. (*d*) The North error $\omega_{\max}$, the half bandwidth *δ*_*ω*_ of $\omega_{\max}$ and the sum $\Sigma =\omega_{\max}^{\mathrm{morning}}+\omega_{\max}^{\mathrm{afternoon}}$ of morning and afternoon North errors during the summer solstice (Sol: 21 June) and the spring equinox (Equ: 21 March).

To determine the North error Δ*ω*_*North*_ (figure 5) derived from the error function *e*(*p*) measured for a cordierite, tourmaline or calcite crystal, we chose 10 real sky situations with different cloud covers meaning 10 different weather situations (figure 6*a*–*c*). The patterns of the degree of polarization *p* of these skies were measured in the green (550 nm) part of the spectrum with a full-sky imaging polarimeter (developed by Estrato Research and Development Ltd., Budapest, [45]) functioning on the top of a building of the Astronomical Observatory of Baja (southern Hungary, 46°10′48.5^′′^ N, 19°00′39.0^′′^ E).

Using the error function *e*(*p*) (figures 3 and 4) and the 10 sky situations (figure 6*a*–*c*), our self-written computer program computed the error Δ*ω*_North_ of the estimated Northern direction relative to the true North angle *ω*_North_ in the following way:

(1) During data processing, we excluded the sky areas with *p*<5% (being under the sensitivity threshold of the human eye) or *p*>90% (that normally does not occur in real skies). These excluded celestial regions were not considered in our further computations.
(2) We chose point pairs (*m*_1_, *m*_2_) from the non-excluded sky areas as follows: (i) The first point *m*_1_ was chosen from a celestial quadratic grid, the square cells of which had a side length of 20 pixels (figure 5*a*). (ii) The second point *m*_2_ was chosen from a celestial polar grid with 15° resolution running between angular distances 45°≤*ρ*≤90° from *m*_1_ (figure 5*b*). According to our earlier field experience with sunstones [13,20,37], point *m*_2_ cannot be too near (0°<*ρ*<45°) or too far (90°<*ρ*≤180°) from *m*_1_, otherwise the accuracy of the second step of sky-polarimetric Viking navigation decreases considerably (see the steps of sky-polarimetric Viking navigation described in the Introduction).
(3) Using the *p* patterns of the 10 measured sky situations (figure 6*a*–*c*), we determined the degrees of polarizations *p*_1_ and *p*_2_ in sky points *m*_1_ and *m*_2_, and calculated the errors *e*_1_=*e*(*p*_1_) and *e*_2_=*e*(*p*_2_) of sunstone adjustment using the measured error function *e*(*p*) of a given analyser (figures 3 and 4).
(4) Let *C*_1*E*_ and *C*_2*E*_ be the great circles passing through the sunstone centres *m*_1_ and *m*_2_ parallel to the straight markings engraved into the sunstone surface during calibration. The estimated sun position *E* is the intersection of circles *C*_1*E*_ and *C*_2*E*_ (figure 5*c*). Let *C*_1*S*_ and *C*_2*S*_ be the celestial great circles connecting the sun *S* with points *m*_1_ and *m*_2_ (figure 5*d*). For each member *m*_*i*_ of the point pair *m*_1_ and *m*_2_, we considered the two great circles *C*_*i*+_ and *C*_*i*−_ enclosing an angle of 2*e*_*i*_(*p*_*i*_) with each other around the great circle *C*_*iS*_ connecting points *m*_*i*_ and *S*, where *i*=1, 2. *C*_*i*+_ and *C*_*i*−_ enclose an angle of +*e*_*i*_(*p*_*i*_) and −*e*_*i*_(*p*_*i*_) with *C*_*iS*_, respectively (figure 5*d*). The intersections of circles *C*_1+_, *C*_1−_ and *C*_2+_, *C*_2−_ appoint a spherical tetragon (marked with grey in figure 5*d*) involving the real sun position *S*. Due to the maximum errors ±*e*_*i*_(*p*_*i*_) of sunstone adjustments, all possible estimated sun positions *E* are placed within this grey tetragon for the given point pair *m*_1_ and *m*_2_. There were maximum *M*_1_⋅*M*_2_=972 000 such celestial tetragons possible for one situation, where *M*_1_=900 and *M*_2_=1080 were the number of sky points *m*_1_ and *m*_2_ in our study.
(5) From the estimated position *E* of the invisible sun, the Viking navigator derived the direction (angle) *ω*_North_ of the geographical North with the use of the well-known sun-compass as follows (figure 5*e*): he might have determined the direction of the imaginary light rays originating from *E* with a shadow-stick [13,20]. If there were no errors of the sunstone adjustment (*e*_1_=*e*_2_=0), the tip of the gnomon shadow would fall on the appropriate gnomonic line engraved in the disc of the Viking sun-compass, and the mirror symmetry axis of the gnomonic line would point towards the geographical North *ω*_North_. Because of inaccurate sunstone adjustments (*e*_1_≠0,*e*_2_≠0), the shadow tip may not fall on the gnomonic line. Then, the sun-compass disc should be rotated with angle Δ*ω*_North_ around its vertical axis so that the shadow tip falls onto the gnomonic line. This angle Δ*ω*_North_ is the navigational (or compass) error belonging to a specific pair of sky points *m*_1_ and *m*_2_, errors *e*_1_ and *e*_2_ of sunstone adjustment, a given point *E* of the grey celestial tetragon (figure 5*d*), and a given date. We divided the grey celestial tetragon involving all possible estimated sun positions *E* into 400 separate points with a uniform distribution. Δ*ω*_North_ was calculated for all of these 400 points of the grey tetragon.
(6) The navigational errors Δ*ω*_North_ were collected into a histogram, which were smoothed (convoluted) by a Gaussian function with a width of 5° at half maximum. This smoothed curve represented the distribution of the North error Δ*ω*_North_ with a maximum at angle $\omega_{\max}$ and a half bandwidth *δ*_*ω*_ meaning the full width at half maximum (figure 5*f*). The smaller the $\left|\omega_{\max}\right|$ and *δ*_*ω*_, the more accurate the sky-polarimetric Viking navigation.
(7) There are always two possibilities to project the estimated sun position *E* onto the gnomonic line (figure 5*g*): one in the morning (when the sun-compass disc is rotated until the shadow tip falls on the morning half of the gnomonic line) and another in the afternoon (when the sun-compass disc is rotated until the shadow tip falls on the afternoon half of the gnomonic line). Thus, we split these two cases and determined the navigation errors Δ*ω*_North_, and the derived parameters $\omega_{\max}$ and *δ*_*ω*_ for morning and afternoon separately.

In all investigated situations, we chose only such solar elevations *θ*_S_ that could have occurred in the onetime Viking habitats at the 61° northern latitude. Furthermore, we considered only two specific dates (figure 5*h*): the spring equinox (21 March) and the summer solstice (21 June), to which the two extrema of the gnomonic line belong. Thus, the maximal solar elevation was below 29°, which is the highest angular distance of the sun from the horizon during the equinox. The gnomonic lines were calculated with the program developed by Bernáth *et al.* [14]. The highest possible solar elevation *θ*_S_ was calculated from the standard astronomical formula [46]:

2 $$\sin\theta_{S}=\sin\phi \cdot \sin\delta +\cos\phi \cdot \cos\delta \cdot \cos\tau ,$$

where *ϕ* is the geographical latitude, *δ* is the right ascension angle and *τ* is the hour angle (which is 0° in the case of maximal elevation).

## 3. Results

### 3.1 Dichroic cordierite and tourmaline sunstones

#### 3.1.1 Adjustment error of cordierite and tourmaline

According to figure 2, both the mean *μ* and the median *ε* of the adjustment errors of dichroic cordierite and tourmaline approached zero as the degree of polarization *p* of incident light increased from 0 to 100% in experiment 1. The deviation of |*μ*| from zero was the smallest for the cordierite: at *N* = 12 depolarizers (producing transmitted light with *p*=5.5%), the mean adjustment error was only |*μ*|=3°, which was the maximum, while for the tourmaline, we obtained |*μ*|=9.4°. The summed absolute values of the mean errors *μ* for both crystals were |*μ*|^cordierite^_sum_=12.9° for the cordierite and |*μ*|^tourmaline^_sum_=20.9° for the tourmaline, whereas the summed absolute values of the medians *ε* were |*ε*|^cordierite^_sum_=13° and |*ε*|^tourmaline^_sum_=24°. The sense of calculating these summed absolute values is the following: after we evaluated the results of the 100 series of measurements, we obtained 100 individual data for each *p* value used in our experiment. Considering the average of these data for individual *p* values, it is difficult to judge the accuracy of the crystals in general. Thus, we introduced the summed absolute values of the mean *μ* and median *ε* values, which is calculated by adding up these values for the two crystals, so that we could quantitatively decide which crystal was better. Hence, considering |*μ*|_sum_ and |*ε*|_sum_, the cordierite was better than the tourmaline.

The standard deviations *Σ* of adjustment errors of dichroic cordierite and tourmaline increased with decreasing *p* of incident light (figure 2). To determine the threshold p* below which the test persons could not sense the periodical intensity change of light transmitted through these dichroic analysers, we performed the following: assuming that if *p*<p*, the test persons adjusted randomly (with equal probability) the orientation of the analysers, we obtained Σ*=45.0° for both the tourmaline and cordierite crystals. In the cases of the tourmaline and cordierite, *Σ* was smaller than the threshold Σ*. Figure 3 displays the error function

3 $$e(p)=\frac{1^{\circ }}{a\cdot p+b}+c,\;\text{with }0\leq p_{\min}<p<p_{\max}\leq 1,$$

of the adjustment of cordierite and tourmaline crystals, for which the obtained numerical values of parameters *a*, *b*, *c*, $p_{\min}$ and $p_{\max}$ are given in table 2.

**Table 2.** Numerical values of the error function *e*(*p*)=(1°/(*a*⋅*p*+*b*))+*c* with $p_{\min}<p<p_{\max}$ obtained for the adjustment of cordierite, tourmaline and four calcite sunstone crystals. Electronic supplementary material, table S1, contains the numerical values of the standard deviations Δ*a*, Δ*b* and Δ*c* of the fitting parameters *a*, *b* and *c*, respectively.

sunstone crystal	*a*	*b*	*c*	$p_{\min}$	$p_{\max}$
cordierite	+0.004222	+0.01173	+0.554°	0.05	0.900
tourmaline	+0.002666	+0.01029	−0.669°	0.05	0.900
calcite 1 (equal intensity: task 1)	*e*(*p*)=+22.5°			0.05	0.369
calcite 1 (equal intensity: task 1)	+0.000421	+0.01315	−11.362°	0.369	0.900
calcite 1 (maximal contrast: task 2)	+0.001062	+0.02831	−3.047°	0.050	0.900
calcite 2 (equal intensity: task 1)	*e*(*p*)=+22.5°			0.050	0.071
calcite 2 (equal intensity: task 1)	+0.012731	−0.06262	+6.493°	0.071	0.900
calcite 2 (maximal contrast: task 2)	+0.006524	+0.01753	+4.716°	0.050	0.900
calcite 3 (equal intensity: task 1)	+0.011235	−0.03914	+6.537°	0.050	0.900
calcite 3 (maximal contrast: task 2)	+0.029276	−0.05855	+13.301°	0.050	0.900
calcite 4 (equal intensity: task 1)	+0.002941	+0.02531	+2.578°	0.050	0.900
calcite 4 (maximal contrast: task 2)	+0.002519	+0.03505	+1.638°	0.050	0.900

#### 3.1.2 Degree of polarization thresholds of intensity change perception in cordierite and tourmaline

Table 1 contains the fraction *f* of the total number of cases (*n* = 100) when the test persons could not sense intensity changes through the rotating cordierite and tourmaline as functions of the number *N* of depolarizers (between the polarizing dial and the analysers) and the degree of polarization *p* (%) of transmitted light in the green (550 nm) part of the spectrum in which the human eye is the most sensitive. In fraction *f*≥0.1 (cordierite) and *f*≥0.2 (tourmaline), the test persons lost the signal (i.e. could not detect the intensity variations) if the number of depolarizers was *N*≥9. However, in fraction *f* = 0.03 (cordierite) and *f*=0.02 (tourmaline), no signal was detected already at *N*=7 (*p*_green_=20.6%). With increasing *N*, more and more test persons with increasing fraction *f* could not see the signal: for *N*=9, 11 and 12 (*p*_green_≤11.4%), the fraction *f* at which the signal was lost using tourmaline ( *f*=0.20, 0.58, 0.64) was approximately twice as high as when cordierite ( *f*=0.10, 0.31, 0.39) was used (table 1).

#### 3.1.3 North error for cordierite and tourmaline

Figure 6*d* shows the accuracy of North determination derived from the measured error functions *e*(*p*) of the cordierite and tourmaline (figure 3) calculated for 10 different sky conditions (figure 6*a*–*c*) that are characterized by the average *p*_ave_ and maximum $p_{\max}$ of the degree *p* of sky polarization calculated for the non-excluded sky regions. The accuracy is characterized by the North error $\omega_{\max}$, the half bandwidth *δ*_*ω*_ of $\omega_{\max}$ (figure 5*f*) and the sum $\Sigma =\omega_{\max}^{\mathrm{morning}}+\omega_{\max}^{\mathrm{afternoon}}$ of the morning and afternoon North errors during the summer solstice (21 June) and the spring equinox (21 March). The sense of calculating *Σ* is the following: The morning $\left(\omega_{\max}^{\mathrm{morning}}\right)$ and afternoon $\left(\omega_{\max}^{\mathrm{afternoon}}\right)$ North errors have an opposite sign (+/−) and their value may differ from zero considerably (figure 5*g*). A Viking navigator, in all probability, corrected the seafaring direction several times throughout the morning and the afternoon. Due to their opposite sign, the accumulation of the morning and afternoon errors can result in a small net error *Σ*, the consequence of which is an accurate navigation on daily average [12]. Table 3 contains the net navigation error $\Sigma =\omega_{\max}^{\mathrm{morning}}+\omega_{\max}^{\mathrm{afternoon}}$ for the most cloudy sky 1 and the least cloudy sky 10 in figure 6 at summer solstice (21 June) and spring equinox (21 March). From figure 6*d*, the following tendencies can be read as functions of the sky conditions:

(i) The half bandwidth *δ*_*ω*_ of $\omega_{\max}$ has a decreasing tendency with increasing *p*_ave_ and $p_{\max}$ for both dichroic cordierite and tourmaline crystals. The sum $\Sigma =\omega_{\max}^{\mathrm{morning}}+\omega_{\max}^{\mathrm{afternoon}}$ of morning and afternoon North errors are around zero in most of the cases. Furthermore, both the North errors $\omega_{\max}$ and their half bandwidths *δ*_*ω*_ at the summer solstice $\left(\omega_{\max}=-39^{\circ }-24^{\circ },\delta_{\omega }=22^{\circ }-90^{\circ }\right)$ have practically the same values as those at the spring equinox $\left(\omega_{\max}=-38^{\circ }-27^{\circ },\delta_{\omega }=23^{\circ }-90^{\circ }\right)$.
(ii) The ranges of North errors and their half bandwidths were narrower for the cordierite $\left(\omega_{\max}=-30^{\circ }-27^{\circ },\delta_{\omega }=22^{\circ }-75^{\circ }\right)$ than for the tourmaline $\left(\omega_{\max}=-39^{\circ }-26^{\circ },\delta_{\omega }=29^{\circ }-90^{\circ }\right)$.

**Table 3.** The net navigation error $\Sigma =\omega_{\max}^{\mathrm{morning}}+\omega_{\max}^{\mathrm{afternoon}}$ for the most (sky 1 in figure 6) and least (sky 10 in figure 6) cloudy sky at summer solstice (21 June) and spring equinox (21 March). $p_{\max}=\text{maximum}$ of the degree of sky polarization calculated for the non-excluded sky regions.

		$\Sigma =\omega_{\max}^{\mathrm{morning}}+\omega_{\max}^{\mathrm{afternoon}}$		
sky in figure 6	1: overcast		10: almost clear	
$p_{\max}$ (%)	38.04		77.25	

	solstice	equinox	solstice	equinox
cordierite	−10°	−10°	−1°	−1°
tourmaline	−15°	−17°	0°	0°

### 3.2 Birefringent calcite sunstones

#### 3.2.1 Adjustment error of calcite

The measured mean *μ* and the median *ε* of the adjustment errors of the four calcite crystals approached zero as the degree of polarization *p* of incident light increased from 0 to 100% both in the equal intensity (task 1) and maximal contrast (task 2) cases. This finding is in accordance with the result obtained for dichroic cordierite and tourmaline crystals. For the contaminated calcites 1 and 2, the minimal *p* value at which the test persons could still find equal intensity was 36.9 and 7.1%, respectively.

The standard deviation *Σ* of calcite adjustment errors increased with decreasing *p* of incident light in both adjustment tasks for all four calcite crystals. This is also in accordance with the result obtained for dichroic cordierite and tourmaline sunstones.

We compared the summed absolute values *Σ*|*μ*| and *Σ*|*ε*| of the mean *μ* and median *ε* of the adjustment errors (table 4). Due to the contaminations of calcites 1 and 2, *Σ*|*μ*| and *Σ*|*ε*| were calculated for 36.9%≤*p*<100%. For task 1 (equal intensity), calcites 1 and 4 were the worst and the best, respectively, considering both the mean and the median of adjustment errors (table 4). Calcites 2 and 3 were similarly accurate, what is not surprising, since they were split from the same major calcite crystal. However, surprisingly, calcite 3 with polished surfaces was slightly less accurate than the unpolished calcite 2 (table 4). This unexpected effect of polishing is addressed later in the Discussion.

**Table 4.** Summed absolute values *Σ*|*μ*| and *Σ*|*ε*| of the mean *μ* and median *ε* of the adjustment errors cumulated for degrees of linear polarization 36.9%<*p*<100% in the case of the four studied calcite crystals for task 1 (equal intensity adjustment) and task 2 (maximal contrast adjustment).

	*Σ*|*μ*|(°)		*Σ*|*ε*| (°)	
analyser	equal intensity (task 1)	maximal contrast (task 2)	equal intensity (task 1)	maximal contrast (task 2)
calcite 1	20.4	4.3	26.0	4.0
calcite 2	11.4	8.4	11.5	10.0
calcite 3	14.0	16.4	16.5	8.0
calcite 4	5.0	3.6	8.5	5.5

For task 2 (maximal contrast), the most accurate crystal was calcite 4, while the least accurate was calcite 3 (table 4). For calcites 1, 2 and 4, the values of *Σ*|*μ*| and *Σ*|*ε*| for task 2 were smaller than those for task 1. This suggests that the maximal contrast (task 2) was easier to detect even at lower *p* values than the equal intensity (task 1), furthermore, the different contaminations in the optical paths do not shift the angle of maximal contrast, independently of *p*.

The error functions of the four calcite crystals for equal intensity (task 1) and maximal contrast (task 2) adjustment are shown in figure 4 and table 2. Note that we did not use sky areas with *p*<5% and *p*>90% for North error determination.

#### 3.2.2 Degree of polarization thresholds of intensity change perception in calcite

The maximal contrast adjustment method (task 2) was more comfortable for the test persons (electronic supplementary material, Result S1), and thus, a navigator could use it more confidently. In 35% of all the 2200 measurements, in task 2 the degree of polarization below which intensity changes could not be detected between the light spots seen in all four calcite sunstones was p*=11.4%, while for calcite 1 in task 1 (equal intensity adjustment) it was p*=20.6%. On the other hand, in 4% of all the 2200 measurements, p* < 10% for all four calcites in both tasks with the exception of calcite 1 in task 1 where p* = 20.6%.

#### 3.2.3 North error for calcite

**Figure 7.**
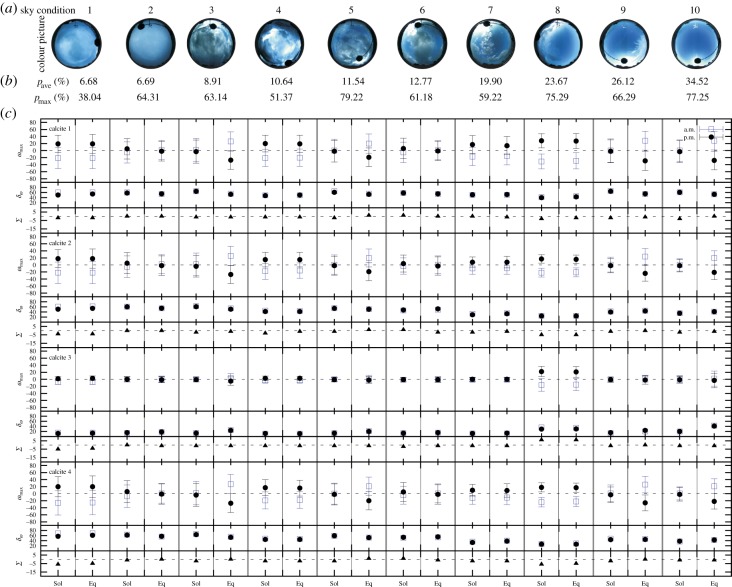
Accuracy of the North determination derived from the measured error function *e*(*p*) of the four different calcite crystals for task 1 (equal intensity adjustment) calculated for 10 different cloudy sky conditions measured by full-sky imaging polarimetry. (*a*) 180° field-of-view colour picture of the sky photographed under different weather conditions without a polarizer. (*b*) The average *p*_ave_ and maximum $p_{\max}$ of the degree of linear polarization *p* calculated for the non-excluded sky regions with 5%<*p*<90%. (*c*) $\omega_{\max}$, *δ*_*ω*_ and $\Sigma =\omega_{\max}^{\mathrm{morning}}+\omega_{\max}^{\mathrm{afternoon}}$ at the summer solstice Sol (21 June) and the spring equinox Eq (21 March).

**Figure 8.**
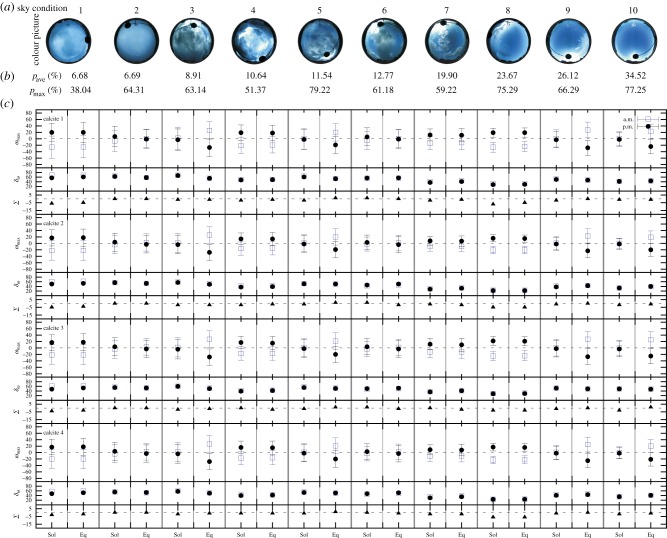
As figure 7 for the maximal contrast adjustment of calcite crystals (task 2).

From the measured error functions *e*(*p*) in figure 4, equation (3) and table 2, we determined the North error Δ*ω*_North_ for the 10 different sky conditions in figures 7*a* and 8*a* that are characterized with the average *p*_ave_ and maximum $p_{\max}$ of the degree of sky polarization *p* calculated for the non-excluded sky regions with 5%<*p*<90%. The distribution of the North error Δ*ω*_North_ has its maximum at angle $\omega_{\max}$ and with a half bandwidth *δ*_*ω*_ meaning the full width at half maximum (figure 5*f*). We determined $\omega_{\max}$, *δ*_*ω*_ and $\Sigma =\omega_{\max}^{\mathrm{morning}}+\omega_{\max}^{\mathrm{afternoon}}$ for the summer solstice (21 June) and the spring equinox (21 March) separately for the equal intensity (task 1, figure 7) and maximal contrast (task 2, figure 8) adjustments of calcite sunstones. The following general tendencies were found: at higher *p*_ave_ and $p_{\max}$, the half bandwidth *δ*_*ω*_ of North errors is usually lower and the daily net error *Σ* is close to zero (−7°<*Σ*<+6°) in all cases. Under less cloudy weather conditions, *δ*_*ω*_ is usually smaller.

Table 5 shows the range (minimum and maximum) of Δ*ω*_North_ and *δ*_*ω*_ of the four calcite crystals: in task 1 (equal intensity adjustment), at solstice Δ*ω*_North_ was slightly smaller than at equinox, while the *δ*_*ω*_ values were similar in both seasons. Calcites 2 and 3 were more accurate (having narrower ranges of Δ*ω*_North_) than calcites 1 and 4. In addition, calcite 1 was the worst (possessing the wider range of Δ*ω*_North_ and the greatest minimum of *δ*_*ω*_). In task 2 (maximal contrast adjustment), the ranges of Δ*ω*_North_ for summer solstice were generally lower than those for spring equinox, while the *δ*_*ω*_ values were similar. Interestingly, in task 2, calcite 3 had a wider range (minimum–maximum) of Δ*ω*_North_ and *δ*_*ω*_ than in task 1 (equal intensity adjustment). For the other three calcite crystals, the tendency was the opposite: the boundary values were slightly lower and the ranges were narrower in task 2 compared to task 1.

**Table 5.** Ranges (minimum–maximum) of the North error (Δ*ω*_North_) and half bandwidth (*δ*_*ω*_) obtained in North error determination for task 1 (equal intensity adjustment) and task 2 (maximal contrast adjustment) for the four studied calcite crystals at summer solstice (21 June) and spring equinox (21 March).

	Δ*ω*_North_ (°)				*δ*_*ω*_ (°)			
	solstice		equinox		solstice		equinox	
analyser	equal intensity (task 1)	maximal contrast (task 2)	equal intensity (task 1)	maximal contrast (task 2)	equal intensity (task 1)	maximal contrast (task 2)	equal intensity (task 1)	maximal contrast (task 2)
calcite 1	−31 to +28	−26 to +20	−36 to +35	−28 to +28	27–66	18–69	27–60	19–68
calcite 2	−22 to +21	−22 to +20	−27 to +26	−28 to +26	20–63	18–61	20–62	19–62
calcite 3	−23 to +22	−28 to +27	−32 to +31	−37 to +36	11–52	26–62	11–50	27–62
calcite 4	−26 to +20	−23 to +19	−27 to +27	−28 to +27	18–69	17–60	20–69	18–60

Table 6 shows the net navigation error $\Sigma =\omega_{\max}^{\mathrm{morning}}+\omega_{\max}^{\mathrm{afternoon}}$ for the most (sky 1 in figures 7 and 8) and the least (sky 10 in figures 7 and 8) cloudy sky in task 1 (equal intensity adjustment) and task 2 (maximal contrast adjustment) for the four studied calcite crystals at summer solstice (21 June) and spring equinox (21 March).

**Table 6.** Net navigation error $\Sigma =\omega_{\max}^{\mathrm{morning}}+\omega_{\max}^{\mathrm{afternoon}}$ for the most (sky 1 in figures 7 and 8) and the least (sky 10 in figures 7 and 8) cloudy sky in task 1 (equal intensity adjustment) and task 2 (maximal contrast adjustment) for the four studied calcite crystals at summer solstice (21 June) and spring equinox (21 March). $p_{\max}=\text{maximum}$ of the degree *p* of sky polarization calculated for the non-excluded sky regions.

	$\Sigma =\omega_{\max}^{\mathrm{morning}}+\omega_{\max}^{\mathrm{afternoon}}$							
sky in figures 7 and 8	1: overcast				10: almost clear			
$p_{\max}$ (%)	38.04				77.25			
	solstice		equinox		solstice		equinox	
analyser	equal intensity (task 1)	maximal contrast (task 2)	equal intensity (task 1)	maximal contrast (task 2)	equal intensity (task 1)	maximal contrast (task 2)	equal intensity (task 1)	maximal contrast (task 2)
calcite 1	−2	−6	−2	−5	−3	−1	0	−1
calcite 2	−4	−5	−4	−4	−2	−2	−1	−1
calcite 3	−5	−4	−4	−3	−1	−3	−1	+1
calcite 4	−6	−3	−5	−2	−1	−2	−1	−1

## 4. Discussion

Viking navigators might had many years of navigational experience. Three of our test persons who previously had participated in navigational field experiments with calcite sunstones [14] generally had a better performance in our present experiments. It is rather trivial that an experienced navigator can use the sunstones more accurately than our test persons. Thus, in our experiments, the error of sunstone adjustment was overestimated, because the majority of our test persons were unexperienced in such an orientation task. On the other hand, however, using the polarization optically ideal analysers in our experiments, the error of sunstone adjustment was underestimated, because Viking navigators might had optically unideal sunstone crystals, and they used these sunstones under the harsh conditions of voyages. The consequence of the latter is the degradation of performance outdoors, in the open air with bright sky and clouds and many other objects in the field of view. These over- and underestimations of the error of sunstone adjustment weaken each other, and nobody knows which effect is stronger.

The adjustment errors of cordierite were smaller than those of tourmaline, furthermore the North determination was more accurate for cordierite than for tourmaline. Thus, cordierite seems to be a slightly better sunstone than tourmaline.

From the summed absolute values of mean errors and the results of North determination, comparing calcite 2 (unpolished) and calcite 3 (polished), we can see that the polished calcite had worse results. At first, this might be surprising. As polishing makes the crystal more transparent, it can be assumed to be easier to detect the intensity differences between the two light spots. However, due to the increased transparency caused by the surface polishing, the internal crystal defects can become more visible, which can confuse and mislead the navigator and result in adjusting the sunstone to a wrong orientation. On the other hand, using a calcite with dull surfaces, the navigator can see the average intensity of the polarized light passing through the crystal, while the local contaminations are blurred. Calcite crystals found in nature are always contaminated, full of inner and superficial scratches, impurities and defects. Hence, according to our present result, it is advisable not to polish the surface of calcite sunstones.

In the case of dichroic cordierite and tourmaline crystals used in experiment 1, the task was always to find intensity minimum, thus crystal contaminations did not disturb the test persons’ accuracy. Hence, it was enough to measure only one sample of both dichroic crystals.

When we compared the two adjustment methods (finding equal intensity or maximal contrast of the two light spots seen in calcite), we found that the results of calcite crystals 1, 2 and 4 were generally better in the case of maximal contrast adjustment than those for the equal intensity adjustment in the same situations. Only the polished calcite 3 was worse concerning both the summed absolute mean error and North determination when the task was to find the maximal contrast.

The test persons marked less cases when they did not see any intensity changes while rotating the calcite crystals for the maximal contrast adjustment (task 2) than for the equal intensity adjustment (task 1). As we used the same settings in the two adjustment tasks, the results should have been similar and independent of the calcite contamination. We assume that the test persons in task 1 (equal intensity adjustment) may also have marked those cases when they could not adjust the crystal into the equal intensity angle (since there was no such angle due to calcite contamination). Thus, false markings coming from the misinterpretation of the task (signalling the cases where no intensity difference can be detected) cannot be exluded. This could not occur when the maximal contrast orientation of calcite had to be found. Hence, we regard the maximal contrast adjustment as a more suitable method for navigation, because misinterpretations cannot occur here and we obtained 240 less markings than in equal intensity adjustment.

A Viking navigator possibly used two calcite sunstones, which could easily be of different qualities. Obviously, the navigation is the most accurate if two identical crystals of the best calcite (calcite 4 in our measurement) with the smallest errors are used, while the navigation is the least accurate if two identical crystals of the worst calcite (calcite 1 in our measurement) with the largest errors are used (figure 4). The navigation accuracies of all other calcite combinations would fall between these two extremes.

As in most cases, the deviations of the North error $\omega_{\max}$ from zero were very similar in the morning and the afternoon, and differed only in the sign, their sum *Σ* always being close to zero (−7°<*Σ*<+6°). This means the following: if a Viking navigator estimated the North direction with the sky-polarimetric method several times during a day (approximately uniformly distributed in the morning and the afternoon) and adjusted the sailing direction accordingly, the net navigation error with which the route deviated from the expected direction was around zero. Hence, many measurements can result in nearly zero North error on average throughout the day. Our results also showed that the half bandwidth *δ*_*ω*_ of the North error $\omega_{\max}$ decreases as the average degree of sky polarization *p*_ave_ and $p_{\max}$ increases. This means that under skies with less clouds (with higher *p*_ave_ and $p_{\max}$) the sky-polarimetric Viking navigation is more accurate. Under less cloudy skies, less measurements/estimations of the North direction are enough to keep the right sailing direction.

Our results also suggest that very thick fog or cloud layers (producing skylight with *p*_ave_<10% in situations 1–3 in figures 6–8) considerably hinders the sky-polarimetric Viking navigation, because then the half bandwidth *δ*_*ω*_ of the North error $\omega_{\max}$ is very high, and in some cases the sum *Σ* of the morning and afternoon North errors differs significantly from zero.

From the North determination results, it turned out that navigation at the summer solstice (21 June) is more accurate than at spring equinox (21 March). This result is beneficial for summertime navigation, because the length of the days around the solstice is much longer than around the equinoxes, so the Vikings had more time to travel on the ocean, to correct bearing several times during a day, and thus the net error could consist of more measurements. Consequently, in a real navigational situation, the net error could be even smaller at summer solstice.

## 5. Conclusion

(1) Based on the results of our psychopysical laboratory experiments and the computation of the North error determination, we conclude that both cordierite and tourmaline crystals are appropriate for functioning as a dichroic sunstone in the first step of sky-polarimetric Viking navigation.
(2) We found that the threshold p* of the degree of polarization below which the periodic intensity change of a rotating sunstone cannot be detected by the naked eye is about 21% for both tourmaline and cordierite; furthermore, the used cordierite had generally better results (regarding |*μ*|_sum_, |*ε*|_sum_, $\omega_{\max}$ and *δ*_*ω*_) than the tourmaline.
(3) Considering the summed absolute values of the mean |*μ*| and median |*ε*| of the adjustment error angles, the applicability of cordierite for sky-polarimetric Viking navigation was about twice as good as that of tourmaline.
(4) The applicability of calcite as a sunstone depends strongly on its optical properties. It is advisable to choose calcite crystals with as few contaminations in the optical path (slots/spots) as possible. We showed that real calcite crystals are not so ideal sunstones as it had been believed earlier, because they usually contain contaminations (scratches, impurities, crystal defects) which increase considerably their adjustment errors.
(5) We experienced that it is not advisable to polish calcite sunstones, since due to their clearer surface the contaminations become more visible, which disturbs and deceives the navigator. The intensity of light in the two slots/spots seen by the navigator should be as homogeneous as possible.
(6) We observed that calcite sunstones have smaller adjustment errors if the navigator looks for the orientation of the crystal where the intensity difference between the two slots/spots seen in the crystal surface is maximal (maximal contrast adjustment), rather than minimal (equal intensity adjustment).
(7) We found that for higher degrees of polarization *p* (>*p*_crit_) of incident light, the adjustment errors of calcite are larger than those of the dichroic cordierite (*p*_crit_=20%) and tourmaline (*p*_crit_=45%) crystals, while for lower *p* (<*p*_crit_), calcite usually has lower adjustment errors than dichroic sunstones (figure 4).
(8) Calcite crystal 1 was the most contaminated, and this resulted in its worst performance: it had larger adjustment errors than the other three calcites as well as the cordierite and tourmaline studied.
(9) Hence, real calcite crystals also have disadvantages, and thus cordierite and tourmaline can also be at least as good sunstones.
(10) The net navigation error *Σ* was between −3° and +1° for almost clear skies and between −17° and −2° for totally overcast skies, where *Σ*=0° means the geographical northern direction. Under clear meteorological conditions, using calcite or cordierite or tourmaline sunstones, Viking sailors could navigate with net orientation errors $|\Sigma_{\max}|\leq 3^{\circ }$. Under overcast conditions, the net navigation error depends on the sunstone type: $|\Sigma_{\max}(\text{calcite})|\leq 6^{\circ }$, $|\Sigma_{\max}(\text{cordierite})|\leq 10^{\circ }$ and $|\Sigma_{\max}(\text{tourmaline})|\leq 17^{\circ }$. According to our findings, in situations when the zenith is clear (not covered by fog or clouds), the first step of sky-polarimetric Viking navigation can be accurate enough to keep the sailing direction, if the other two steps are errorless.
(11) Our computations of the errors of North determination derived from the error functions of dichroic and birefringent sunstone crystals in the first step of sky-polarimetric Viking navigation showed that this method can be reliable under clear skies (with high average degrees *p*_ave_ and $p_{\max}$ of sky polarization), but it becomes inaccurate under foggy or overcast conditions (with low average *p*_ave_ and $p_{\max}$).

## Supplementary Material

Electronic Supplemetary Material for Adjustment errors of sunstones in the first step of sky-polarimetric Viking navigation: Studies with dichroic cordierite/tourmaline and birefringent calcite crystals

## Data accessibility

All supporting data from our studies are made available as the electronic supplementary material of this paper.
