# Supplementary material for: Adjustment errors of sunstones in the first step of sky-polarimetric Viking navigation: studies with dichroic cordierite/ tourmaline and birefringent calcite crystals
Source: R Soc Open Sci. 2016 Jan 20;3(1):150406. doi: 10.1098/rsos.150406 (PMC4736922; doi:10.1098/rsos.150406)
Supplement: Electronic Supplemetary Material for Adjustment errors of sunstones in the first step of sky-polarimetric Viking navigation: Studies with dichroic cordierite/tourmaline and birefringent calcite crystals [file rsos150406supp1.doc]

**Electronic Supplemetary Material**

**for**

**Adjustment errors of sunstones in the first step of sky-polarimetric**

**Viking navigation: Studies with dichroic cordierite/tourmaline**

**and birefringent calcite crystals**

Dénes Száz, Alexandra Farkas, Miklós Blahó, András Barta, Ádám Egri, Balázs Kretzer,

Tibor Hegedüs, Zoltán Jäger and Gábor Horváth^1,*^

1. Environmental Optics Laboratory, Department of Biological Physics, Physical Institute,

Eötvös University, H-1117 Budapest, Pázmány sétány 1, Hungary

*Corresponding author: gh@arago.elte.hu

This file contains the following: Supplementary Result S1

Supplementary Table S1

Supplementary Figures S1, S2

**Supplementary Result S1**

**Degree of Polarization Thresholds of Intensity Change Perception in Calcite**

We marked those cases when the test persons did not sense at all the intensity change of light transmitted through the rotating calcite. We examined the numbers of these cases at degrees of polarization *p* = 36.9, 20.6, 11.4, 7.1, 5.5 %. For higher *p*-values the test persons could always detect intensity changes while rotating the crystals. Altogether 1077 cases were counted when no intensity changes were detected (Supplementary Fig. S2). Concerning the numbers of these cases, there was a difference between the equal intensity (task 1) and maximal contrast (task 2) adjustments of calcite (Supplementary Fig. S2): In task 1 (equal intensity) 240 more cases were marked than in task 2 (maximal contrast). This suggests that the maximal contrast adjustment method was more comfortable for the test persons, and a navigator could use it more confidently. Determining a threshold line at which no intensity changes were detected in 10 % of the measurements at a given *p*-value, for all calcites but calcite 1 in task 1 and all calcites in task 2, the degree of polarization below which intensity changes could not be detected between the light spots seen in calcite was *p** = 11.4 %, while for calcite 1 in task 1 it was *p** = 20.6 %. These cases took 35 % of all the 2200 measurements (10 *p*-values for 11 test persons repeated 10 times in 2 tasks, 10 × 11 × 10 × 2 = 2200). For setting the above threshold to 50 %, *p** < 10 % for all calcites in both tasks with the exception of calcite 1 in task 1 where *p** = 20.6 %. These cases took 4 % of all the 2200 measurements (Supplementary Fig. S2).

**Supplementary Table S1**: Numerical values of the standard deviations Δ*a*, Δ*b* and Δ*c* of the fitting parameters *a*, *b* and *c* for the error function $e(p)=\frac{1^{o}}{a\cdot p+b}+c$ with *p*_min_ < *p* < *p*_max_ obtained for the adjustment of cordierite, tourmaline and four calcite sunstone crystals.

| **sunstone crystal** | **±Δ*a*** | **±Δ*b*** | **±Δ*c*** | ***p*_min_** | ***p*_max_** |
| --- | --- | --- | --- | --- | --- |
| **cordierite** | 0.000918 | 0.0048 | 0.001^o^ | 0.050 | 0.900 |
| **tourmaline** | 0.000686 | 0.00351 | 0.0173^o^ | 0.050 | 0.900 |
| **calcite 1**  **(equal intensity: task 1)** | 0.000044 | 0.0061 | 0.1329^o^ | 0.369 | 0.900 |
| **calcite 1**  **(maximal contrast: task 2)** | 0.00052 | 0.00198 | 0.0378^o^ | 0.050 | 0.900 |
| **calcite 2**  **(equal intensity: task 1)** | 0.0066 | 0.0046 | 0.1821^o^ | 0.071 | 0.900 |
| **calcite 2**  **(maximal contrast: task 2)** | 0.000394 | 0.00206 | 0.1826^o^ | 0.050 | 0.900 |
| **calcite 3**  **(equal intensity: task 1)** | 0.00154 | 0.00834 | 0.7082^o^ | 0.050 | 0.900 |
| **calcite 3**  **(maximal contrast: task 2)** | 0.00125 | 0.00684 | 0.5455^o^ | 0.050 | 0.900 |
| **calcite 4**  **(equal intensity: task 1)** | 0.000966 | 0.00438 | 0.1532^o^ | 0.050 | 0.900 |
| **calcite 4**  **(maximal contrast: task 2)** | 0.000178 | 0.00676 | 0.3144^o^ | 0.050 | 0.900 |


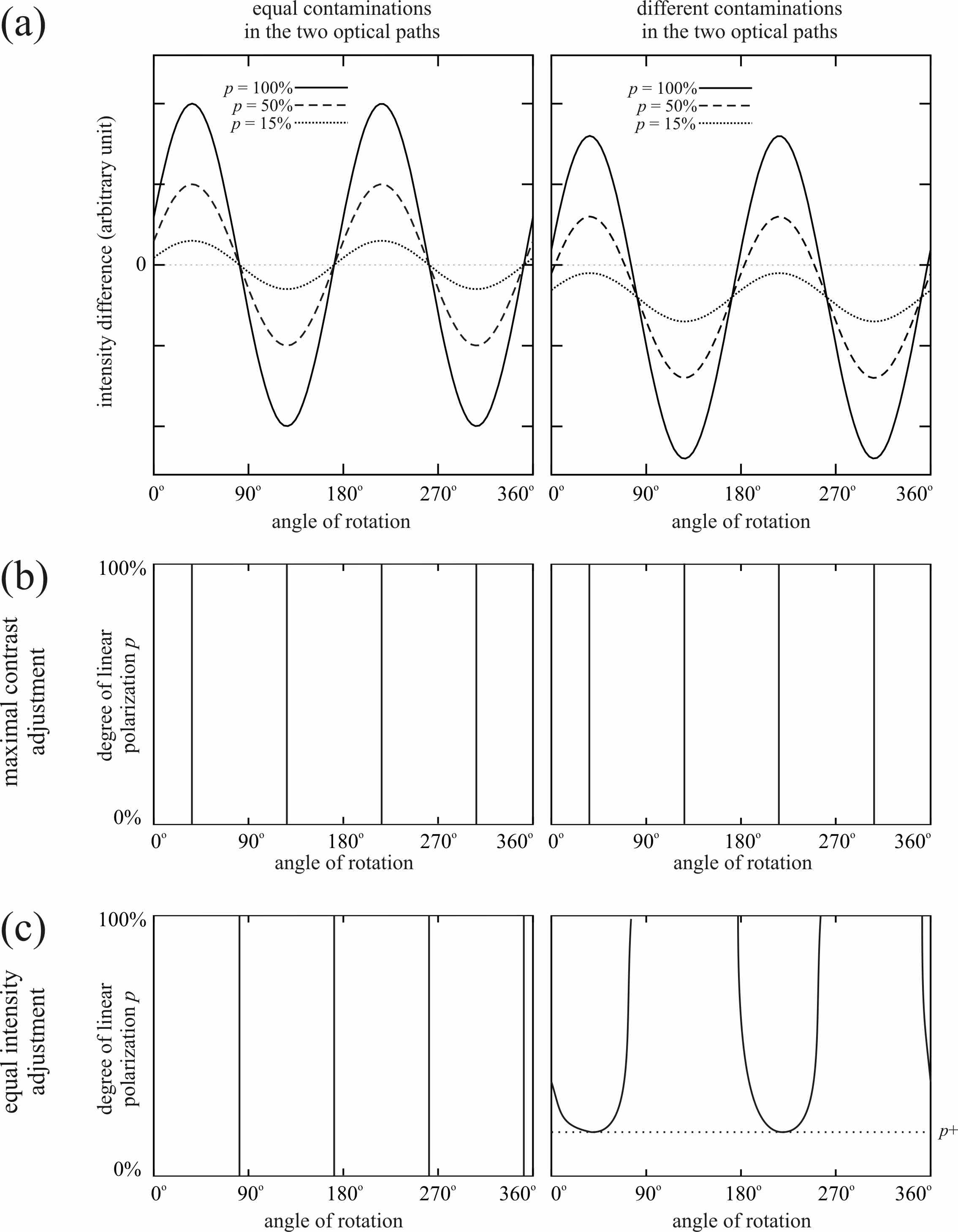


**Supplementary Figure S1:** Demonstration of the effect occurring because of the different contaminations (scratches/impurities/defects) in the two optical paths of a calcite crystal. (a) Intensity difference between the two light spots seen in the rotating calcite illuminated by white light with different degrees of linear polarization *p* if the contaminations are equal (left) and different (right) in the two optical paths. (b) Maximal intensity difference orientation of the calcite if the contaminations are equal (left) and different (right) in the two optical paths. (c) Equal intensity difference orientation of the calcite if the contaminations are equal (left) and different (right) in the two optical paths. Calcite crystals with different contaminations have a threshold *p*^+^ of the degree of polarization, under which (*p* < *p*^+^) no equal intensity of the two light spots can be detected.


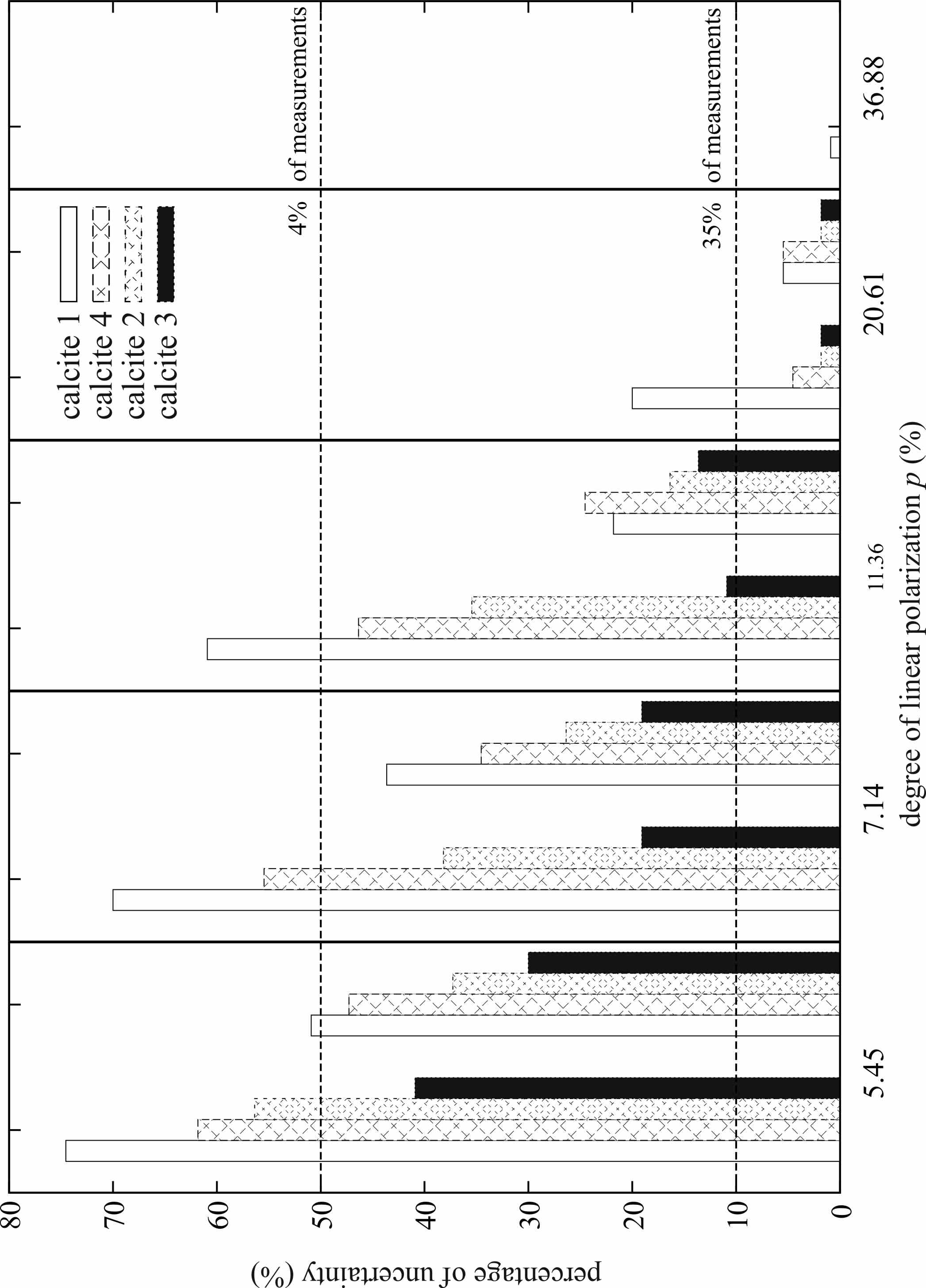


**Supplementary Figure S2:** Percentage of uncertainty among the 110 measurements in experiment 2 for task 1 (equal intensity adjustment) and task 2 (maximal contrast adjustment) when the test persons could not sense intensity changes in the light spots of the rotating calcite crystals as a function of the degree of linear polarization *p* (%) of incident white light. The horizontal dashed line marks the thresholds at 10 % and 50 % of uncertainty (the percentage of the measurements for a given crystal and a given *p*-value, when no intensity changes were detected), above which the sky-polarimetric Viking navigation cannot be used. At the 10 % threshold 35 % of all the 2200 measurements was regarded as insecure, while at the 50 % threshold, only 4 % of all the 2200 measurements were insecure.
